# Seasonal Influenza: A Narrative Review of Epidemiology, Clinical Features, and Preventive Strategies

**DOI:** 10.7759/cureus.95336

**Published:** 2025-10-24

**Authors:** Yousif Kyokha Ameen

**Affiliations:** 1 College of Medicine, University of Sulaimani, Sulaymaniyah, IRQ

**Keywords:** infectious disease, influenza, influenza vaccine, internal med, vaccine

## Abstract

Seasonal influenza, caused by influenza A and B viruses, remains a significant global respiratory illness, particularly during winter epidemics. Transmission occurs mainly through respiratory droplets containing high viral loads expelled during coughing or sneezing. This review aims to summarize recent developments in the clinical features, prevention, and evolving treatment strategies for seasonal influenza. Relevant literature from global and regional (including South Asian) sources was reviewed to identify updates in influenza epidemiology, diagnosis, vaccination, and antiviral therapy. Influenza typically presents with sudden-onset fever, myalgia, headache, and malaise, accompanied by upper respiratory tract symptoms such as sore throat, rhinorrhea, and nonproductive cough. While most healthy adults clear the virus within five days, children, the elderly, and immunocompromised individuals may shed it for up to 10 days or longer. Severe complications involving the brain, heart, or lungs may lead to hospitalization. Annual vaccination remains the most effective preventive strategy, while early antiviral therapy, initiated within 48 hours of symptom onset, reduces morbidity and mortality. Despite advances in prevention and management, the global burden of influenza persists. Strengthening surveillance, improving vaccine coverage, and promoting timely antiviral use are essential to reduce seasonal influenza-related morbidity and mortality.

## Introduction and background

Introduction

Influenza, commonly known as “the flu,” is an acute viral infection caused by influenza viruses that primarily target the respiratory tract. It remains a major cause of morbidity and mortality worldwide, with an estimated one billion cases and up to 650,000 respiratory deaths annually, according to the World Health Organization (WHO) [1].

Transmission occurs mainly through respiratory droplets expelled during coughing or sneezing, typically requiring close contact for infection. Most healthy individuals recover within a week; however, severe disease and complications can develop among high-risk groups such as young children, pregnant women, the elderly, and those with chronic illnesses or immunosuppression [2].

Unlike many other respiratory viruses, the influenza virus rapidly spreads globally due to frequent antigenic variation, enabling the emergence of novel strains. These antigenic shifts and drifts drive seasonal epidemics and occasional pandemics, underscoring the virus’s ongoing threat to public health [3].

This review summarizes the epidemiology, clinical features, and complications of influenza and highlights recent advances in its diagnosis, management, and prevention.

History

Influenza epidemics have been recorded for centuries, typically occurring every one to four years in different regions of the world. The first confirmed outbreak was documented in 1694, although descriptions of influenza-like illness trace back to 1173-1174 [4,5].

The 1918-1919 H1N1 “Spanish flu” pandemic remains the most catastrophic, causing an estimated 50 million deaths globally, far higher than early 20th-century reports [6]. Later pandemics included the 1957 “Asian flu” (H2N2), the 1968 “Hong Kong flu” (H3N2), and the 2009 “swine flu” (H1N1pdm09) [7]. These events exemplify the influenza virus’s ability to undergo antigenic shift and genetic reassortment, leading to the emergence of novel subtypes with pandemic potential.

In recent decades, novel avian-origin influenza subtypes such as H5N1 and H7N9 have demonstrated the ongoing potential for zoonotic transmission and pandemic emergence, emphasizing the need for continued genomic surveillance and molecular characterization of viral evolution [8].

**Figure 1:**
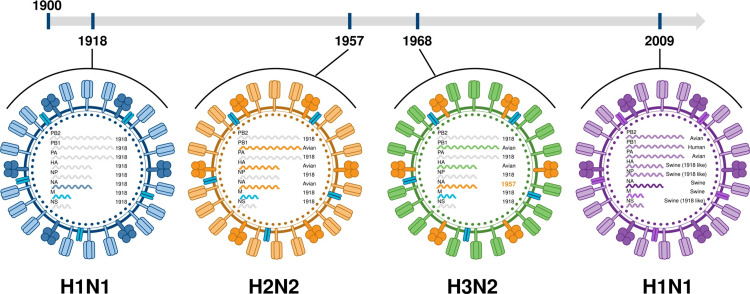
History of influenza pandemics Reproduced from [7]. Licensed under the Creative Commons Attribution 4.0 International License (CC BY 4.0). This license permits unrestricted use, distribution, and reproduction in any medium, provided the original author and source are credited, a link to the license is provided, and any changes made are indicated.

Figure 1 illustrates the major influenza pandemics of the 20th and 21st centuries (1918 H1N1, 1957 H2N2, 1968 H3N2, and 2009 H1N1pdm09), emphasizing the molecular mechanisms, particularly gene segment reassortment, that produced each pandemic strain [9].

Etiology

Influenza viruses are enveloped, single-stranded, negative-sense RNA viruses belonging to the Orthomyxoviridae family. They are classified into three main types (A, B, and C) based on their internal nucleoprotein and matrix protein antigens. Types A and B are responsible for most influenza epidemics, whereas type C typically causes mild upper respiratory illness [10].

The influenza virion is roughly spherical or filamentous in shape. It contains a lipid envelope that is embedded with two key glycoproteins, hemagglutinin (HA) and neuraminidase (NA), which are essential for viral entry and release, respectively. The genome comprises eight RNA segments encoding at least 11 proteins, allowing high genetic variability through antigenic drift (point mutations) and antigenic shift (gene reassortment) [10].

To date, at least eighteen HA (H1-H18) and eleven NA (N1-N11) subtypes of influenza A have been identified, resulting in combinations such as H1N1 and H3N2. The virus nomenclature also incorporates host species, geographic origin, strain number, and year of isolation. Influenza B lacks distinct subtypes but has evolved into two antigenically distinct lineages, B/Yamagata and B/Victoria, since the 1970s [11].

Emerging avian influenza strains, such as H5N1 and H7N9, have demonstrated the virus’s capacity for cross-species transmission and molecular adaptation through mutations in HA receptor-binding sites and polymerase genes, underscoring the ongoing evolutionary threat posed by influenza A variants [12].

Although influenza B and A have a comparable viral structure, the absence of subtypes in influenza B is attributed to the virus's fixed antigenic features, specifically HA and NA. Despite this, the virus has begun to divide into two antigenically distinct lineages, and there have been reports of minor antigenic variabilities in this virus since 1970 [13].

## Review

Epidemiology

Influenza epidemics of varying intensity occur annually across all regions, influenced by viral transmissibility, population immunity, and antigenic characteristics. The global burden remains substantial; according to the WHO, influenza causes approximately one billion infections, 3-5 million cases of severe illness, and 290,000-650,000 deaths worldwide each year. These patterns are shaped by seasonal fluctuations and the virus’s continuous genetic evolution [1].

During the 2022-2023 influenza season, vaccine effectiveness was estimated at around 50%, offering higher protection against influenza A (H3N2) and H1N1 strains but somewhat lower protection against influenza B. Despite imperfect strain matching, vaccination remained effective in preventing severe disease and hospitalization [14]. Recent WHO FluNet data highlight regional differences in circulating strains, emphasizing the importance of ongoing genomic surveillance and timely vaccine updates [15].

Influenza A viruses evolve through two primary mechanisms of antigenic variation: drift and shift. Antigenic drift involves gradual point mutations in HA and NA genes, leading to minor antigenic changes and recurring seasonal outbreaks of varying intensity [16]. In contrast, antigenic shift results from major genetic reassortment between animal and human strains, producing novel subtypes capable of triggering pandemics [17]. The virus’s segmented RNA genome facilitates these reassortment events, contributing to the emergence of pandemic strains such as H1N1 (2009) and H3N2 (1968) [17].

The burden of influenza is unevenly distributed across populations. Children experience the highest infection rates, while severe complications and deaths are more common among the elderly and individuals with comorbidities such as cardiovascular disease, diabetes, or chronic respiratory conditions. Pregnant women, particularly in the second and third trimesters, face an increased risk of complications and mortality due to physiological and immunologic changes [18,19]. Overall, global surveillance data continue to underscore influenza’s capacity for rapid evolution and widespread transmission, reinforcing the need for annual vaccination and adaptive public health strategies (Table 1) [20].

**Table 1:** Groups at higher risk of complications from influenza HIV: human immunodeficiency

Category	Examples/notes
Infants	Unvaccinated children aged 12–24 months
Chronic respiratory diseases	Asthma, cystic fibrosis (children), chronic obstructive pulmonary disease (adults)
Cardiovascular disease	Persons with hemodynamically significant cardiac disorders
Immunosuppression	Individuals with immunosuppressive disorders or receiving immunosuppressive therapy, people with HIV infection
Hematologic disorders	Sickle-cell anemia and other hemoglobinopathies
Long-term aspirin therapy	Children or adolescents with chronic diseases (e.g., rheumatoid arthritis) requiring prolonged aspirin treatment
Renal dysfunction	Persons with chronic kidney disease
Malignancy	Individuals undergoing treatment for cancer
Metabolic disorders	Diabetes mellitus and other chronic metabolic conditions
Neurologic or neurodevelopmental disorders	Seizure disorders, neuromuscular conditions, or cognitive impairment that may compromise handling of respiratory secretions
Older adults	Adults aged ≥65 years
Residents of congregate settings	Persons of any age living in nursing homes or other long-term care institutions

Transmission

Transmission occurs mainly via respiratory droplets (>5 μm) expelled during coughing or sneezing, which generally travel short distances and settle quickly, thus requiring close contact for infection [21]. In addition to droplet spread, poor hand hygiene significantly contributes to transmission, particularly in children. Practices such as wiping or touching the nasal canal with contaminated handkerchiefs or hands can facilitate indirect contact transmission, an often underrecognized but important route for influenza spread.

Although droplet transmission has long been considered the dominant route, recent evidence indicates that aerosolized particles can also play a major role. Studies have demonstrated that fine aerosols containing viable influenza virus can remain airborne for extended periods and contribute substantially to community spread. In one 2023 analysis, aerosol transmission accounted for nearly 50% of influenza transmission events, suggesting that infection control strategies focusing solely on droplet precautions may be insufficient [21-22].

Certain groups, including young children, immunocompromised individuals, the elderly, and patients with chronic diseases, tend to shed the virus for longer periods and remain infectious for extended periods, thereby increasing the risk of secondary transmission [23]. Overall, influenza transmission is best understood as multimodal, involving droplets, aerosols, and contact with contaminated surfaces, with relative contributions varying by environment and host factors.

Clinical manifestations

Influenza symptoms typically begin abruptly after an incubation period of one to two days; however, this period may vary depending on the viral strain. Certain strains have a longer incubation period before symptom onset, while others, particularly more transmissible or virulent variants, can cause symptoms to appear more rapidly. Systemic features such as fever, rigors, headache, acute myalgia, malaise, and anorexia are predominant, with severity often linked to the intensity of fever and myalgia [24]. Myalgia commonly affects the calf, paravertebral, and back muscles but can involve any striated muscle, including the extraocular muscles, leading to painful eye movements. Respiratory symptoms, such as a dry cough, rhinorrhea, and sore throat, usually accompany these systemic signs. Because the onset is abrupt, patients often recall the exact time their illness began. Most influenza cases are self-limited febrile illnesses, though severe systemic disease can occur in vulnerable groups [25].

In pediatric patients, influenza can present with both respiratory and extra-respiratory manifestations. While fever and cough remain common, gastrointestinal symptoms such as vomiting and diarrhea are more frequent than in adults. Otitis media is a typical complication, and febrile seizures may occur in younger children. Importantly, children may shed the virus for longer periods and sometimes appear less systemically ill despite being highly infectious, contributing significantly to transmission within households and schools [26].

Complications of influenza

Influenza infection can lead to a wide range of complications involving the respiratory, cardiovascular, muscular, and neurological systems.

Primary viral pneumonia typically develops soon after a classic influenza episode and presents with high fever, dyspnea, cyanosis, and hemoptysis. It is more common in individuals with underlying heart or lung diseases, such as asthma or chronic obstructive pulmonary disease. Imaging often reveals bilateral reticular or reticulonodular opacities, sometimes with consolidation [25]. Radiologically, it may resemble pulmonary edema, particularly in the lower lobes, where perihilar congestion and hazy opacification are observed [27]. Commonly used severity indices such as CURB-65 or the Pneumonia Severity Index are not validated for influenza-related pneumonia; thus, hospitalization decisions rely on clinical judgment, especially in cases of hypotension, pregnancy, or, in children, low oxygen saturation, tachypnea (commonly defined as >25 breaths/min in adults, though the threshold varies with age, typically higher in children and lower in the elderly), or concurrent diarrhea. In severe cases, multilobar infiltrates and acute respiratory distress syndrome can develop rapidly within two to five days of symptom onset, with respiratory failure often occurring within 24 hours of hospitalization and requiring intubation and mechanical ventilation [28].

Secondary bacterial pneumonia develops in approximately 10-15% of hospitalized influenza cases, particularly among elderly patients and those with underlying comorbidities [29]. The most frequent pathogens are *Streptococcus pneumoniae* and *Staphylococcus aureus* (including community-acquired MRSA) [30,31]. Typically, patients show initial improvement followed by relapse with renewed fever, productive cough, and dyspnea, accompanied by new radiographic consolidations. This biphasic clinical course strongly suggests bacterial superinfection [31].

Beyond the lungs, influenza can also cause systemic effects. Cardiac involvement may include myocarditis and pericarditis, rare but potentially serious complications, with a significant proportion showing ECG abnormalities [32]. Most cases tend to resolve over weeks to months, with many patients regaining normal cardiac function and exhibiting only transient abnormalities [33].

Neurological complications are very rare but may include encephalitis, encephalopathy, seizures, and Guillain-Barré syndrome [34]. In children, additional complications are relatively frequent. These include acute otitis media, febrile seizures, and gastrointestinal symptoms such as vomiting and diarrhea. Rarely, influenza-associated encephalopathy or Reye syndrome may occur, especially in those exposed to salicylates during illness [35].

Diagnosis

Clinical manifestations remain the cornerstone of influenza diagnosis, and laboratory investigations are not routinely required for most patients. However, confirmatory testing is warranted in cases with atypical presentation, hospitalization, or high-risk comorbidities. Laboratory confirmation is primarily achieved through nucleic acid amplification tests (e.g., PCR), rapid antigen assays, or, less commonly, virus culture.

Rapid influenza diagnostic tests detect viral antigens in respiratory specimens within approximately 15-30 minutes, allowing timely results for clinical decision-making. Most can differentiate between influenza A and B, though they cannot identify subtypes such as H1N1 or H3N2 [36]. While specificity remains consistently high, sensitivity varies widely (4-80%) depending on patient age, viral load, and specimen type [37-39]. Studies indicate that sensitivity tends to be higher in children and early in the course of illness when viral titers peak. Although cost-effectiveness analyses are mixed, point-of-care testing combined with targeted antiviral therapy may be cost-effective in many clinical settings [38,39].

Molecular methods, particularly polymerase chain reaction (PCR), have become the standard for influenza detection in hospital-based laboratories due to their superior sensitivity and ability to simultaneously identify virus types and subtypes by amplifying multiple genetic targets [25]. Compared with viral culture, PCR can detect nonviable viral RNA, enabling earlier diagnosis, and nasopharyngeal swabs generally yield the most reliable results [25,40]. Advances such as multiplex PCR panels now enable the detection of influenza alongside other respiratory pathogens. At the same time, genomic sequencing enhances surveillance by monitoring antiviral resistance and identifying emerging variants in real time [40].

In most otherwise healthy individuals, diagnostic testing is unnecessary, as the disease is typically self-limiting. Testing becomes important when results are expected to influence management, such as guiding antiviral therapy decisions, distinguishing viral from bacterial infections to reduce unnecessary antibiotic use, implementing infection control measures, or confirming influenza in hospitalized patients with fever and severe respiratory illness, including those with community-acquired pneumonia [25]. Appropriate use of diagnostic tools not only supports individual case management but also strengthens epidemiological surveillance and informs public health strategies during influenza outbreaks.

Medical treatment

The U.S. Food and Drug Administration currently approves four antiviral drugs for the treatment and prevention of influenza: oseltamivir, zanamivir, peramivir, and baloxavir marboxil. While most healthy individuals recover without medication, antivirals can reduce disease duration and complications, particularly when started within 48 hours of symptom onset [41]. Early initiation offers the most significant benefit, but treatment may still be effective for severe or high-risk cases when initiated later [25].

Antiviral treatment is recommended for patients hospitalized with confirmed or suspected influenza, individuals at high risk for complications (Table 1) with suspected or confirmed influenza within 48 hours of symptom onset, and high-risk outpatients with persistent or worsening symptoms, even if testing occurs >48 hours after onset.

Neuraminidase inhibitors

NA inhibitors (NAIs), oseltamivir and zanamivir, remain the first-line agents against most influenza A and B viruses. They act by blocking the NA enzyme, inhibiting viral release and spread. During the 2009 H1N1 pandemic, NAIs were widely used and demonstrated reductions in mortality and ventilator use when started early [42]. Sporadic oseltamivir resistance has been observed, though most circulating strains remain susceptible; current H3N2 strains show only ~0.2% resistance [43]. Adamantanes are no longer recommended due to widespread resistance among influenza A(H3N2) and A(H1N1)pdm09 strains [44].

Cap-dependent endonuclease inhibitor

Baloxavir marboxil, a novel cap-dependent endonuclease inhibitor, provides an alternative mechanism by blocking viral mRNA transcription. It is administered as a single oral dose and is effective against strains resistant to NAIs. Clinical trials show that baloxavir shortens symptom duration and reduces viral shedding, including in patients with oseltamivir-resistant influenza A and B strains [25,45]. Combination therapy and close monitoring for resistance remain key strategies, particularly in severe or complicated infections [44].

**Table 2:** Suggested dosage and time of antiviral drugs for adult influenza

Antiviral agent	Indication	Recommended dosage (adults)	Pediatric dosage	Duration
Oseltamivir	Treatment	75 mg orally, twice daily	3 mg/kg/dose twice daily (max 75 mg/dose)	5 days
	Chemoprophylaxis	75 mg orally, once daily	3 mg/kg once daily (max 75 mg)	7 days
Zanamivir	Treatment	10 mg (two 5-mg inhalations), twice daily	Not recommended for children <7 years	5 days
	Chemoprophylaxis	10 mg (two 5-mg inhalations), once daily	Not recommended for children <5 years	7 days
Baloxavir marboxil	Treatment	40 mg (body weight 40–<80 kg) or 80 mg (≥80 kg) orally, single dose	2–<12 years: 2 mg/kg (max 40 mg if <80 kg, 80 mg if ≥80 kg), single dose	Single dose
	Chemoprophylaxis	Same as treatment dose	Same as treatment dose	Single dose

Corticosteroids and supportive care

Corticosteroid therapy is not routinely recommended for influenza-related complications, as multiple studies suggest it may increase mortality or delay viral clearance [46,47]. Nevertheless, their use may be justified for specific indications such as refractory septic shock or asthma exacerbations. Supportive management (hydration, oxygen therapy, and monitoring for secondary bacterial infections) remains central to care.

Emerging and experimental therapies

Given the limited range of available antivirals, research is exploring monoclonal antibodies (mAbs) targeting conserved influenza antigens. The M2e (matrix 2 ectodomain) is a particularly promising target due to its conservation across influenza A subtypes [48,49]. Anti-M2e antibodies do not neutralize the virus directly but may act through immune-mediated mechanisms that eliminate infected cells [50,51]. Clinical applications remain experimental, pending further trials. Overall, early antiviral initiation, appropriate patient selection, and resistance surveillance are essential pillars of effective influenza management.

Prevention and chemoprophylaxis

Annual influenza vaccination remains the cornerstone of influenza prevention, offering the most effective protection against infection and severe outcomes [1,52]. Because influenza viruses undergo frequent antigenic changes, new vaccines are reformulated each year to match circulating strains [53]. Vaccine strain selection is based on global surveillance data coordinated by the WHO, which monitors emerging variants and recommends formulations approximately six to eight months before each influenza season: in February for the Northern Hemisphere and September for the Southern Hemisphere [54].

For the 2024-2025 Northern Hemisphere influenza season, the WHO recommends that trivalent vaccines contain the following viral antigens [54]: A/Victoria/4897/2022 (H1N1)pdm09-like virus, A/Thailand/8/2022 (H3N2)-like virus, and B/Austria/1359417/2021 (B/Victoria lineage)-like virus. For quadrivalent vaccines, an additional B/Phuket/3073/2013 (B/Yamagata lineage)-like virus is included. According to the WHO and the U.S. Advisory Committee on Immunization Practices (ACIP), priority groups include pregnant women, children aged 6-59 months, elderly adults (≥65 years), individuals with chronic conditions (e.g., diabetes, renal failure, cardiovascular disease), and healthcare workers and caregivers [55].

The ACIP recommends annual vaccination for all persons aged 6 months and older, unless contraindicated. Children aged six months to eight years receiving the vaccine for the first time should receive two doses, separated by at least four weeks, to ensure an optimal immune response [56].

Influenza activity typically peaks during late fall and winter. In the Northern Hemisphere, vaccination should ideally be completed by October, and in the Southern Hemisphere by May. Children in their first vaccination season require two doses for full protection [57].

The effectiveness of seasonal influenza vaccines depends on the match between circulating and vaccine strains [58]. Antigenic drift can reduce vaccine efficacy, particularly among older adults and immunocompromised populations. During the 2023-2024 Northern Hemisphere influenza season, interim U.S. data indicated an overall vaccine effectiveness of approximately 44% against medically attended influenza illness, with moderate protection across age groups and circulating strains [59].

Nevertheless, even with imperfect matches, vaccination provides cross-protection, reducing severe disease and complications, particularly in high-risk populations [1]. Seasonal influenza vaccines remain safe, well-tolerated, and strongly recommended by the WHO and national health authorities.

Antiviral chemoprevention serves as an adjunct to vaccination, especially for unvaccinated individuals or those with insufficient immune response. NAIs, such as oseltamivir and zanamivir, remain the preferred agents due to their lower global resistance rates than those of adamantanes, though emerging resistance patterns have been observed [44]. Chemoprophylaxis is effective across populations, including residents of long-term care facilities, though no head-to-head comparison of the two agents has demonstrated superiority [25].

The indications for antiviral chemoprophylaxis are the following: influenza outbreaks in long-term care centers among the elderly, regardless of vaccination status; unvaccinated high-risk individuals with close exposure to a confirmed case; and vaccinated individuals at high risk of complications during a season with a poor vaccine match who had close contact within 48 hours of an infected person [25].

The American College of Obstetricians and Gynecologists and the ACIP recommend antiviral prophylaxis for pregnant women and postpartum individuals (within two weeks of delivery) with close contact to individuals with influenza A. Because of its low systemic absorption, zanamivir is preferred in pregnancy when feasible [60,61]. Overall, annual vaccination, complemented by targeted chemoprophylaxis, remains the most effective strategy to reduce influenza morbidity and mortality.

## Conclusions

Influenza continues to impose a significant social and economic burden across all populations, with pregnant women, the elderly, and individuals with chronic conditions at highest risk for severe outcomes. Prevention through timely vaccination remains the most effective public health strategy. Annual influenza vaccination programs should prioritize high-risk groups to reduce hospitalizations and mortality.

While antiviral therapy plays a key role in managing severe cases, its use should be guided by evidence-based protocols to minimize drug resistance. Early initiation, ideally within 48 hours of symptom onset, remains critical, especially for hospitalized and pregnant patients. Strengthening vaccination coverage, ensuring rapid diagnosis, and promoting rational antiviral use are essential policy priorities for reducing the overall impact of influenza.
